# Winter Activity and Dormancy of Snails: Freezing and Food Shortage Avoidance Strategy Facing Snow-Cover Shortage

**DOI:** 10.3390/ani15030348

**Published:** 2025-01-25

**Authors:** Anna M. Lipińska, Zofia Książkiewicz, Adam M. Ćmiel, Oksana Hnatyna, Paulina Laskowska, Dariusz Halabowski

**Affiliations:** 1 Institute of Nature Conservation, Polish Academy of Sciences, Mickiewicza 33, 31-120 Kraków, Poland; cmiel@iop.krakow.pl; 2 Department of General Zoology, Adam Mickiewicz University, Uniwersytetu Poznańskiego 6, 61-614 Poznań, Poland; zofksi@amu.edu.pl; 3 Department of Zoology, Biological Faculty, Ivan Franko National University of Lviv, Hrushevskoho Str. 4, 79005 Lviv, Ukraine; oksana.hnatyna@lnu.edu.ua; 4 Department of General Geology and Geotourism, Faculty of Geology, Geophysics and Environmental Protection, AGH University of Krakow, 30-059 Kraków, Poland; laskowska@agh.edu.pl; 5 Department of Ecology and Vertebrate Zoology, Faculty of Biology and Environmental Protection, University of Lodz, Banacha 12/16, 90-237 Lodz, Poland

**Keywords:** land snails, winter activity, supercooling point (SCP), cold tolerance, shell morphology, environmental gradients, dormancy, snow-cover shortage, adaptive strategies, low-temperature activity

## Abstract

Cold tolerance is a key factor shaping the survival and geographic distribution of terrestrial snails, especially in regions with harsh winters. Understanding how these organisms cope with freezing temperatures is crucial for predicting their responses to changing climates. This study focused on two microsnail species, *Vertigo antivertigo* and *V. moulinsiana*, to assess their winter activity, cold tolerance strategies, and whether their body size varies with latitude. Activity patterns were observed under controlled temperatures (0 °C, 2 °C, and 5 °C), while supercooling points (SCP) were measured to evaluate freezing avoidance. Shell morphology was analyzed across populations from various sites in Poland to explore local adaptations. The results showed that snail activity decreases as temperatures drop, with the lowest activity observed at 0 °C. Both species displayed a freezing-avoidant strategy, with *V. moulinsiana* having slightly higher SCP values, reflecting its adaptation to milder climates. Morphological differences in shell dimensions across sites suggest potential local adaptations to environmental conditions. These findings highlight temperature as a critical driver of activity, survival, and morphological variation in terrestrial snails. Limited winter activity may allow foraging or shelter-seeking but poses risks for overwintering. As climate change leads to snow-free winters, these species may face significant challenges in maintaining their populations and distributions.

## Simple Summary

Cold winters pose significant challenges for land snails, influencing their survival and where they can live. Our study focused on two tiny snail species, *Vertigo antivertigo* and *V. moulinsiana*, to understand how they cope with cold conditions in Poland. We investigated whether these snails stay active during winter, how their body size varies across locations, and how they avoid the freezing of body fluids. We found that snail activity decreases as temperatures drop, with much less movement at 0 °C compared to 2 °C or 5 °C. Both species avoid freezing by lowering their freezing point, but *V. moulinsiana* is slightly less cold-tolerant, likely due to its preference for milder climates. Differences in shell size and shape among populations suggest that local environmental conditions influence their physical traits. While limited winter activity, like finding food or shelter, can be beneficial, it also comes with risks, especially if the snails are not fully prepared for the cold. Our findings emphasize the importance of temperature and snow cover for these snails’ survival. With climate change causing warmer, snow-free winters, these tiny animals may face new challenges in maintaining their populations and adapting to changing environments.

## 1. Introduction

Dormancy is an adaptation that allows organisms to pause development, enabling them to survive unsuitable environmental conditions [1,2,3,4,5,6]. This phenomenon has been documented across almost all animal phyla and numerous taxa, with definitions varying depending on context [6]. Typically, dormancy involves a lowered metabolic rate, reducing energy expenditure. This state of inactivity protects organisms from environmental stressors, such as drought, extreme temperatures, and hypoxia [5,6]. The biochemical mechanisms underlying cold-hardiness and drought resistance overlap with diapause processes, as both involve changes in cellular solute composition, although they are distinct [5,7].

Various theories have been proposed to explain the evolution and widespread occurrence of dormancy [1,5,8,9]. Some focus on phylogenetic explanations, suggesting that dormancy reflects ancestral adaptations [10,11,12,13]. Others highlight environmental drivers, such as the need to survive drought, high temperatures, or cold climates, leading to convergent evolution [1,5,8,9,14,15]. Additionally, dormancy may reduce competition, mitigate food scarcity, or align life cycles with specific environmental conditions [1,5,9,16].

Gastropods, as ectothermic and typically hydrophilic animals, are highly sensitive to temperature and moisture, which regulate their activity and physiology [17,18,19]. In temperate climates, overwintering represents a crucial survival strategy [20,21]. Many land snails bury themselves in litter and soil during winter (e.g., *Helix pomatia*, which buries itself to a depth of 10 cm), while others seek shelter in rotting wood, under tree bark, or in rock crevices [22,23,24,25,26]. Some species, such as the micro-snail *Vertigo moulinsiana*, overwinter in low vegetation and litter [27,28,29].

Shell size in gastropods influences their ability to endure low temperatures and harsh winter conditions [30,31]. Larger shells offer better insulation and may aid in maintaining a stable internal environment during extreme cold [30]. This aligns with the Temperature-Size Rule (TSR), which posits that ectotherms grow larger in cooler environments as an adaptive strategy to optimize thermal regulation and energy efficiency [32,33]. Additionally, shell size impacts the supercooling point (SCP), which is the temperature at which freezing is avoided, through mechanisms like moisture retention and cryoprotectant accumulation. For instance, larger shells may enable greater retention of energy reserves and moisture, facilitating biochemical adjustments critical for supercooling [7,34]. However, smaller-bodied gastropods may employ alternative strategies, such as selecting microhabitats with milder conditions or using behavioral thermoregulation to mitigate freezing risks [24]. Interestingly, some studies have observed snail activity under snow cover [35,36]. Snow acts as an excellent insulator and thermal buffer, protecting organisms from extreme temperature fluctuations. This buffering effect allows ectothermic snails to remain active even when air temperatures above the snow drop below freezing [35,36,37,38]. Our previous research indicates that *Vertigo* snails, particularly *V. moulinsiana*, exhibit a high resistance to low temperatures [28,29]. During overwintering, these snails are typically found on low vegetation and within litter, which is often covered by a layer of snow [27,28,29]. However, questions remain regarding whether these conditions fully suppress their activity during winter.

This study aims to explore whether ectothermic microsnail species *V. antivertigo* and *V. moulinsiana* remain active during winter and whether their shell size correlates with latitude. By analyzing shell size and shape, SCP, and activity at low temperatures across environmental gradients we aim to elucidate how these traits vary among populations and contribute to their cold tolerance strategies. We hypothesized that: (1) snails in colder regions will exhibit larger shell sizes, consistent with the TSR; (2) SCP values will be lower in colder regions; (3) the light/dark cycle will influence snail activity patterns; and (4) snail activity will decrease with declining temperatures. The findings will provide new insights into the ecological effects of temperature on terrestrial microsnails and adaptive responses to climatic variability.

## 2. Materials and Methods

### 2.1. Studied Species

Desmoulin’s whorl snail (*Vertigo moulinsiana* [Dupuy, 1849]) and the marsh whorl snail (*Vertigo antivertigo* [Draparnaud, 1801]) are small European terrestrial snails, with shells measuring approximately 2–3 mm in length. Both inhabit wetlands and marshes, but their conservation statuses differ significantly. *V. moulinsiana* is classified as vulnerable across Europe [39] and is listed in Appendix II of the EU Habitats Directive [40], while *V. antivertigo* is a common and widespread species [41]. Both species exhibit climbing behavior, though with distinct preferences. *V. antivertigo* typically remains near the base of vegetation, while *V. moulinsiana* occupies a broader vertical range and can be found as high as two meters above the ground [42,43,44]. However, in autumn, *V. antivertigo* descends toward the litter where it winters, while *V. moulinsiana* winters on plants [42].

### 2.2. Collection Sites

**Figure 1:**
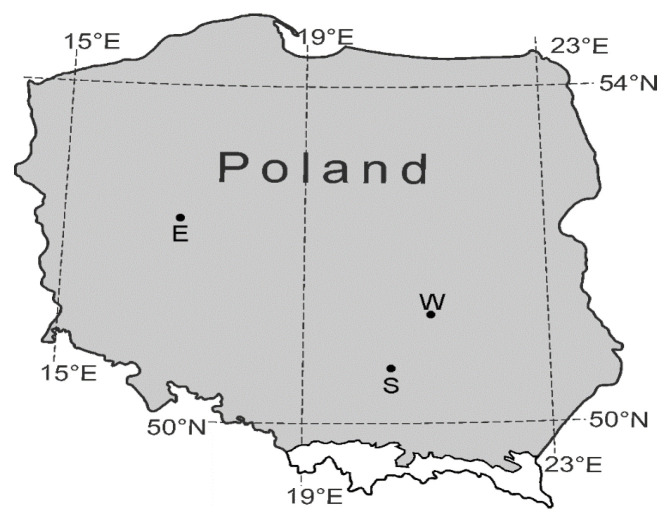
Localization of study sites: east (E), west (W), and south (S). The shaded area indicates the potential distribution range of *Vertigo moulinsiana*. The distribution range of *V. antivertigo* encompasses the entirety of Poland [41].

The study was conducted across three distinct sites in Poland: a wetland area near Kierskie Lake in Poznań (referred to hereafter as the west site), the floodplains of the Nida River in Umianowice near Kielce (the south site), and an extensive wetland area in Pakosław near Radom (the east site; Figure 1). All three sites are located in lowland regions but differ in climatic conditions [45,46]. The west site experiences the mildest climate, with the highest average air temperatures in both summer and winter and the fewest days with snow cover. In contrast, the east site endures in the harshest conditions, featuring the lowest average air temperatures in summer and winter and the highest number of days with snow cover. The south site represents intermediate conditions, characterized by numerous frosty days, very cold weather with precipitation, and relatively few dry cold days (see also Table 1, Figure 1 [45]). Despite being located in floodplains, the south site lacks standing water due to drainage.

### 2.3. Activity Experiment

The experiment comprised two stages: a preliminary study conducted in winter 2022 and a main study in autumn 2022. Both stages shared similar natural photoperiods of 9 h of light and 15 h of darkness (9L:15D). Hourly relative humidity measurements, taken from November to February at the south site consistently indicated very-high humidity levels of 85–95% RH (HOBO Pendant Temperature/Light 64K Data Loggers UA-002-64 HOBO Data Loggers, Bourne, MA, USA; [47]) allowing us to assume that air temperature was the primary factor influencing snail activity.

For the experiments, climate chambers were set to a photoperiod of 12 h of light and 12 h of darkness (12L:12D) with constant air humidity at 85% RH. Non-heat-emitting LED lamps (LDP—LED Domination Power 2W, Aquario, Katowice, Poland) provided illumination. The equal duration of light and dark phases facilitated statistical analyses by ensuring an equal number of observations during both periods. During each experimental trial, snails were placed on glass Petri dishes (100 × 15 mm diameter, five individuals per dish) with six dishes assigned to each temperature treatment. Snails were marked with non-toxic acrylic paint for individual identification. Food and water were provided following the methods used by Cameron [17]. Before observations, snails were acclimated to experimental temperatures of 0 °C, 2 °C, and 5 °C for four days in climate chambers.

In the preliminary study, 180 individuals of *Vertigo moulinsiana* were collected from plant remnants at the south site. After acclimation, snail activity was observed every eight hours for one day. During observations, Petri dishes were briefly removed from the climate chambers (for approximately ten seconds). Activity was assessed under a binocular microscope, and the dishes were immediately returned to the chambers. A snail was classified as active if it was crawling or if its body was extended with tentacles fully everted, following the criteria outlined by Cameron [17].

The main study, conducted in autumn 2022, expanded on the preliminary study by including two species, *V. moulinsiana* and *V. antivertigo*. Individuals were collected from three sites: west, south, and east. For *V. moulinsiana*, 15 individuals were collected from each site. For *V. antivertigo*, 15 individuals were collected from the west and south sites, as this species was absent at the east site. Acclimation procedures were identical to those of the preliminary study, with observations starting on the fifth day and recorded every two hours for two consecutive days. Throughout the experiment, air temperature in the climate chambers remained stable, with deviations not exceeding 1 °C, verified by temperature loggers (HOBO UA-002-64, HOBO Data Loggers, Bourne, MA, USA) placed near the Petri dishes. To prevent heat shock at the conclusion of the experiment, all dishes were first transferred to 5 °C for 12 h and then to 15 °C for another 12 h. Snail activity was checked at each stage. Individuals that remained inactive were transferred to 20 °C for an additional 12 h, after which their activity was assessed again. Snails that remained inactive during these checks and throughout the experiment were considered dead and excluded from further analyses.

### 2.4. Super Cooling Point (SCP) Measurements

According to Sinclair et al. [48], measuring the SCP in 20–30 individuals provides a reliable sample size to determine the SCP distribution. In our experiments, the sample size for each species at each site ranged from 17 to 29 adult individuals with no visible signs of damage or disease. For *V. moulinsiana*, SCP measurements were conducted three times: in winter 2020 [29] and winter 2022, with individuals collected from the south-east site, and in autumn 2022, using individuals collected from all three sites (west, east, and south-east). For *V. antivertigo*, SCP measurements were conducted once, in autumn 2022, using individuals collected from two sites: west and south. The experimental method followed the protocol recommended by Salt [7], previously applied in other studies on land snails [49] and frost resistance in *V. moulinsiana* [29]. A thermocouple (K-type, probe diameter 0.5 mm) was inserted through the shell entrance of each individual. Snails were then placed in a deep freezer (Platilab 340 SV-3-STD; ALS Angelantoni Life Science, Massa Martana, Italy) programmed to decrease the temperature at a constant rate of 1 °C/min [7]. The temperature decrease was continuously monitored, and the SCP was determined from the cooling curve as the upward peak (rebound) caused by the release of heat during crystallization, with an accuracy of ±0.1 °C. After SCP measurement, the snails were gradually warmed to room temperature to assess survival, following the method described by Sinclair et al. [48]. To prevent the heat wave effect and avoid desiccation caused by sudden warming, the temperature was increased gradually at a rate of 1 °C/min.

### 2.5. Shell Size Measurements

**Figure 2:**
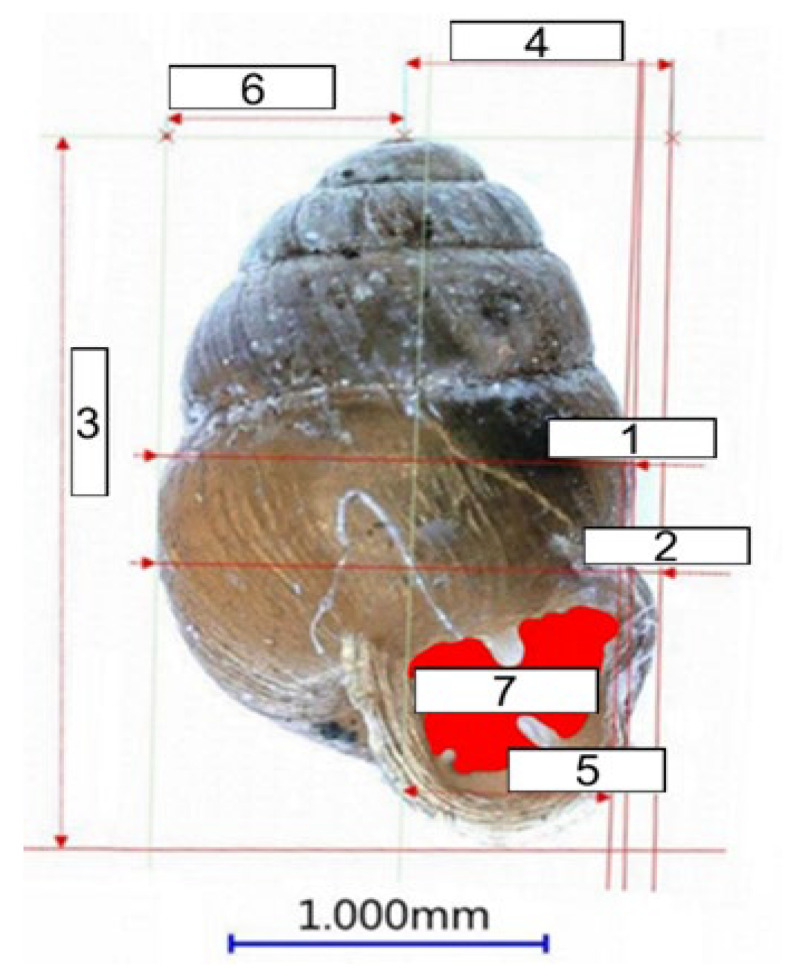
Shell measurements demonstrated on an example of a *Vertigo moulinsiana* individual. 1: width of the last whorl, 2: shell width, 3: shell height, 4: distance from the shell symmetry axis to the outer edge of the shell entrance, 5: shell entrance width, 6: asymmetry of the shell entrance, and 7: shell entrance area.

Shell size measurements were conducted on twenty-eight individuals of *V. moulinsiana* from three sites: nine individuals from the west site, twelve from the east site, and seven from the south site. Additionally, 20 individuals of *V. antivertigo* were measured, including 14 from the west site and 6 from the south site. Measurements were performed using a Keyence VHZ-950F digital microscope, KEYENCE INTERNATIONAL (BELGIUM) NV/SA, Mechelen, Belgia. Only adult individuals with fully developed shells, free from visible signs of damage, were used. Shells were arranged as shown in Figure 2, and all parameters were measured from captured images. Recorded parameters included shell height, shell width at the widest point, width of the last whorl, asymmetry of the shell entrance (distance from the shell symmetry axis to the outer edge), and the surface area of the shell entrance (Figure 2).

### 2.6. Statistical Analysis

The effect of the temperature, locality, and light on the activity of *V. antivertigo* and *V. moulinsiana* snails (autumn study) were analyzed using the repeated measures Generalized Linear Models using IBM SPSS 26.0 software (GLZ; binomial, logit; dependent variable: activity (0/1), categorical variables: temperature, locality, light (0/1), and interactions between categorical predictors: temperature * locality, temperature * light, locality * light).

Homogeneity of variance assumption in SCP and measured shell parameters was tested using Levene’s test. Variables in which variance was not homogenous were transformed using Box–Cox transformation and achieved homogeneity. Therefore, differences in the mean SCP between months and variations in measured shell parameters between sites in *V. moulinsiana*, individuals were tested using a one-way ANOVA. For *V. antivertigo* individuals, differences in the mean SCP between months and shell parameters variations between sites were analyzed using Student’s *t* test.

## 3. Results

### 3.1. Activity Experiments

#### 3.1.1. Preliminary Study

The repeated measures GLZ model revealed that the number of active *V. moulinsiana* individuals was significantly influenced by temperature but not by the light/dark cycle or the interaction between temperature and the cycle (Table 2). Snail activity was significantly lower at 0 °C compared to 2 °C (pairwise contrasts, *p* < 0.001) and 5 °C (pairwise contrasts, *p* < 0.001). However, no significant difference was observed between 2 °C and 5 °C (pairwise contrasts, *p* = 0.203). Notably, at 0 °C, no activity was observed in *V. moulinsiana*.

#### 3.1.2. Main Study

**Figure 3:**
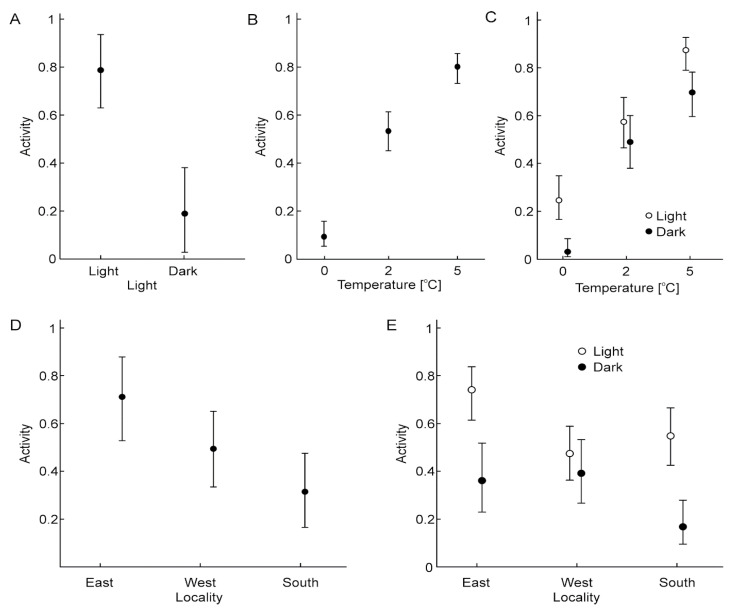
The results of the Generalized Linear Model (GLZ; logit, binomial) showing the effects of the light/dark cycle (**A**), temperature (**B**), the interaction between the light/dark cycle and temperature (**C**), locality (**D**), and the interaction between the light/dark cycle and locality (**E**) on the activity of *Vertigo moulinsiana* individuals.

**Figure 4:**
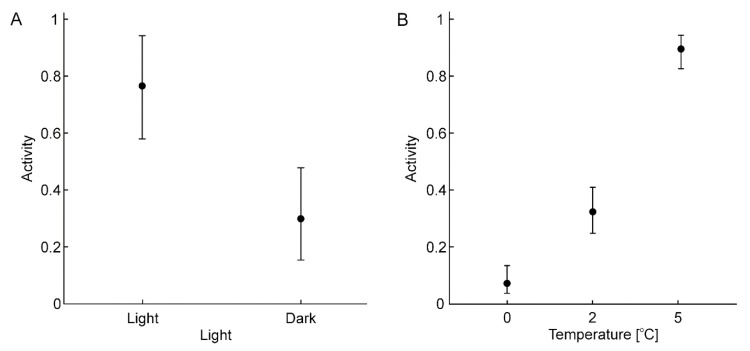
The results of the Generalized Linear Model (GLZ; logit, binomial) showing the effects of light (**A**) and temperature (**B**) on the activity of *Vertigo antivertigo* individuals.

The repeated measures GLZ model demonstrated that temperature, locality, the light/dark cycle, and the interactions between the cycle and temperature, as well as between locality and the cycle, had a significant influence on the activity of *V. moulinsiana* (Table 3). In contrast, the interaction between temperature and locality was not significant (Table 3). The snails were significantly more active during the light phase of the photoperiod (Figure 3A). The highest activity was observed at 5 °C, while the lowest occurred at 0 °C (Figure 3B). Individuals from the east and south-east sites exhibited significantly greater activity during the light phase, whereas this difference was not significant for individuals from the west site (Figure 3C). Interestingly, even at 0 °C, *V. moulinsiana* individuals exhibited greater activity during the light phase (Figure 3D). For *V. antivertigo*, the repeated measures GLZ model showed that only temperature and the light/dark cycle significantly influenced activity (Table 3). Locality and all interactions, including temperature and locality, temperature and cycle, and locality and cycle, were not significant (Table 3). Similar to *V. moulinsiana*, *V. antivertigo* individuals were more active during the light phase of the photoperiod (Figure 4A). The highest activity was recorded at 5 °C, while the lowest occurred at 0 °C (Figure 4B).

### 3.2. Super Cooling Point (SCP) Measurements

Basic statistics for SCP values measured in *V. antivertigo* and *V. moulinsiana* collected at each locality are presented in Table 4. The differences in mean SCP values for *V. moulinsiana* collected at the south site in winter 2020 [29], winter 2022, and autumn 2022 were not significant (one-way ANOVA; F = 2.73; *p* = 0.0724). Similarly, no significant differences were observed in mean SCP values for *V. moulinsiana* collected in autumn 2022 across the three localities (One-Way ANOVA; F = 0.83; *p* = 0.4402). In contrast, the mean SCP value for *V. antivertigo* individuals collected at the south locality was significantly lower than that of individuals collected at the west locality (*t*-test; t = 3.43; *p* = 0.0032).

### 3.3. Shell Size Measurements

**Figure 5:**
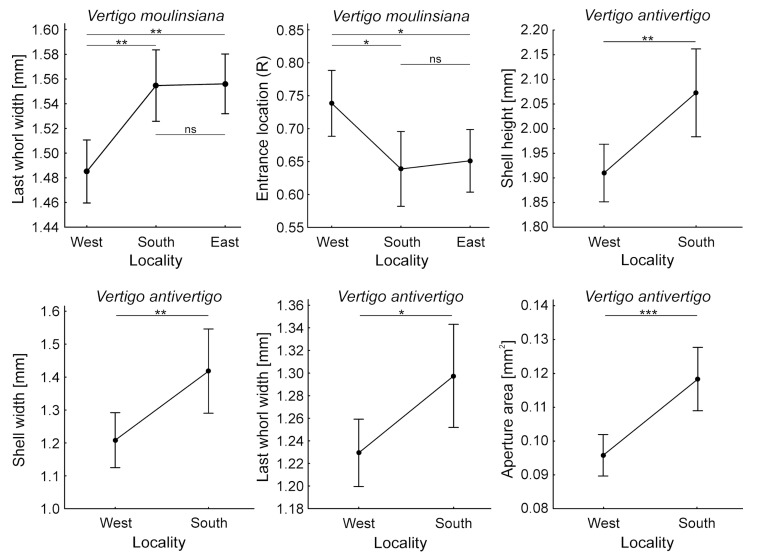
The differences in measured shell parameters between sites for *Vertigo moulinsiana* and *V. antivertigo* were analyzed. For *V. moulinsiana*, the significance of differences was tested using the Unequal N Tukey HSD test (ANOVA post hoc test), while for *V. antivertigo*, the significance was assessed using Student’s *t*-test. Statistical significance is indicated as follows: ns—not significant, *—*p* < 0.05, **—*p* < 0.01, ***—*p* < 0.001.

Basic statistics for shell measurements in *V. antivertigo* and *V. moulinsiana* populations collected from the west, south, and east localities are presented in Table 5. For *V. moulinsiana*, significant differences between the sites were observed in the width of the last whorl (one-way ANOVA; F = 10.68, *p* = 0.0005) and the entrance location (R) (one-way ANOVA; F = 4.82, *p* = 0.0179). However, no significant differences were found in shell height (one-way ANOVA; F = 2.18, *p* = 0.1354), shell width (one-way ANOVA; F = 1.21, *p* = 0.3153), or aperture area (one-way ANOVA; F = 1.6, *p* = 0.2356). Individuals collected from the west site had significantly smaller last whorl widths compared to those from the south and east sites (Figure 5). Additionally, the shell entrance was shifted further to the right in individuals from the west site compared to those from the south and east sites (Figure 5). For *V. antivertigo*, significant differences between sites were detected in shell height (*t*-test; t = 3.2, *p* = 0.0049; Figure 5), shell width (*t*-test; t = 2.89, *p* = 0.0098; Figure 5), last whorl width (*t*-test; t = 2.63, *p* = 0.0171; Figure 5), and aperture area (*t*-test; t = 4.24, *p* = 0.0005; Figure 5). In contrast, the differences in entrance location (R) were not significant (*t*-test; t = 0.76, *p* = 0.4578).

## 4. Discussion

Our study revealed that temperature is a critical factor influencing snail activity, particularly under near-freezing conditions. In both winter and summer experiments, snail activity was significantly lower at 0 °C compared to 2 °C and 5 °C. This trend aligns with previous findings that demonstrate increased snail activity with rising temperatures (e.g., [17,50]). However, earlier research primarily focused on temperatures higher than those tested in this study, making our findings particularly important for understanding activity at near-freezing conditions and providing novel insights into snail behavior at low temperatures.

The obtained results indicated a clear seasonal difference. In the autumn study, some activity of snails was observed at 0 °C. In contrast, during the winter study, snails were not active at this temperature. This difference may be explained by physiological changes between individuals collected in autumn and winter, as the latter were probably already overwintering. Additionally, the results concerning the difference in activity when comparing the light/dark cycle in snails collected in autumn and winter suggest seasonal differences which may result from preparation for overwintering. Specifically, individuals of both species collected in autumn exhibited significantly higher activity during the light phase compared to the dark phase of the experiment. In contrast, this effect was not observed in individuals collected during the winter, further supporting the hypothesis that these snails had already entered a hypometabolic state.

A nocturnal lifestyle is common among snails, particularly in dry environments, where daytime activity increases water loss [17]. Moreover, snails may lose more water under hot conditions than under cold conditions, even at the same saturation deficits [51]. In colder conditions, desiccation is generally less severe, enabling greater flexibility in activity patterns. In other words, colder conditions allow snails to be more active [17]. For example, *Cepaea nemoralis* shows daytime activity only at low temperatures, shifting to nocturnal behavior under milder conditions (especially during nighttime as many other terrestrial snails, which exhibit crepuscular and nocturnal activity) [17]. Both *Vertigo moulinsiana* and *V. antivertigo* used in our study are known for their higher activity in dark conditions [50].

Snail activity under intense illumination in the laboratory may reflect stress responses. In natural environments, increased light exposure often leads to greater water loss due to heat from sunlight. Thus, the activity observed under light conditions in this study could be linked to an active search for shaded shelters that provide protection from light exposure [52]. It is also possible that the behavior of snails in artificial laboratory conditions may not perfectly replicate their natural behavior. Furthermore, the chosen light and dark cycle differs from the natural photoperiod (9L:15D), and this discrepancy could potentially influence snail behavior. Circadian activity in snails can be influenced by various factors, including temperature, wind speed, and the relative humidity of litter moisture [53]. For snails inhabiting the litter, such as species from the *Vertigo* genus, moisture conditions appear to play a greater role in activity patterns than either temperature or light [54]. This suggests that geographic and environmental variability in moisture availability could significantly impact their behavior. Snail activity exhibits diurnal variation, with a stronger response to temperature than to light, and with more intense activity occurring at night than during the day [54]. However, strictly nocturnal activity may be disadvantageous in northern Europe, where summer nights are particularly short [17]. In such conditions, inhabiting the litter may provide significant advantages due to its low light exposure, as well as its buffering properties that help regulate humidity and air temperature [47]. Temperature and moisture conditions vary across environments (environmental variation) and regions (climatic variation), leading to geographic differences in their effects on snail activity depending on the climate zone [19]. However, the significantly lower activity of *V. moulinsiana* individuals from the southern site most likely reflects their poor condition, possibly due to reduced soil moisture and air humidity at this site caused by prolonged drought and a lowered groundwater table. The site is primarily supplied with water through heavy rainfall in July and frequent fogs occurring in summer and autumn [28,55]. Considering the critical role of humidity for snails [19], and especially for *V. moulinsiana* [43,44], the poor condition of individuals from this site may have influenced the experimental results.

Regarding SCP, no significant differences were observed in *V. moulinsiana* across year or sites, suggesting consistent physiological adaptations to low temperatures. In contrast, *V. antivertigo* from the southern site exhibited significantly lower SCP values than individuals from the west, suggesting a regional adaptation. In addition, for both species, the mean SCP value was highest at the west locality, which is characterized by a milder climate. The low SCP values suggest that both snail species employ a “freezing-avoidant” strategy. This strategy has already been described for *V. moulinsiana* [29]; however, similar studies have not yet been conducted for other species of the *Vertigo* genus. Interestingly, *Columella edentula* (SCP: −16.81 °C; [30]) and *C. columella* (SCP: −13.82 °C; [30]) exhibit SCP values comparable to *V. moulinsiana* (min SCP in this study: −11.9 °C). These species share similarities in body size (*V. moulinsiana*: 2.7 mm, *C. edentula*: ~3 mm, and *C. columella*: ~3.5 mm) and occupy similar habitats characterized by very moist to wet conditions on calcareous substrates. *V. moulinsiana* is an Atlantic–Mediterranean species with a distribution ranging from Ireland to Russia and extending south to North Africa [43]. In contrast, *C. edentula* is a Euro–Siberian species, ranging as far as the Caucasus and Central Asia [41]. *C. columella*, an Arctic–Alpine species [56], occurs in northern Europe, the mountains of Central Europe, Siberia, Crimea (Yajla), Armenia, the mountainous regions of the Middle East, and North America. The relatively higher SCP values observed in *V. moulinsiana*, compared to other *Vertigo* species likely reflect its adaptation to milder low-temperature conditions, as opposed to the colder habitats typically inhabited by *Columella* species.

Shell dimensions suggest the distinctiveness of the west site. The shells of *V. antivertigo* from this site were significantly smaller in all measured dimensions, except for the position of the aperture. For *V. moulinsiana*, only the width of the last whorl was significantly smaller at the west site, and the aperture was shifted further to the right compared to shells from the other two sites. Aperture size and position are likely constrained to minimize water loss and protect against parasites and predators [57], yet the exact drivers of geographic variation remain unclear. It is worth noting that differences in the size and appearance of *Vertigo* shells between sites have previously been observed [58]. It is important to acknowledge that the sample sizes for shell measurements (28 individuals for *V. moulinsiana* and 20 for *V. antivertigo*) are relatively small. While these numbers were constrained by the availability of individuals in the studied populations, they still provide valuable insights into the morphological adaptations of these species. Nevertheless, the limited sample sizes may not fully capture the variability across broader populations, and caution is advised when interpreting these findings. Future studies should aim to include larger sample sizes to better account for population-level variability and enhance the generalizability of the results.

The results of our study demonstrate that *V. moulinsiana* and *V. antivertigo* can remain active even at temperatures close to 0 °C. This suggests that snails may remain active or exhibit periodic activity during winter, particularly in mild periods. Additionally, activity beneath the snow cover is also possible. However, the purpose of such winter activity raises important questions. The ability to stay active in winter may provide several benefits. It allows individuals to avoid unfavorable factors such as predators or parasites (e.g., beetles, nematodes, and fly larvae) and to search for better shelters for overwintering. Furthermore, it enables foraging, which could help replenish nutrient deficits, promote faster growth, and lead to earlier maturation in juveniles. However, winter activity also presents significant challenges. Firstly, it conflicts with the physiological preparation for overwintering, a process extensively described in snails [24,30]. This process involves eliminating ice nucleators from the body by clearing the intestines of food debris and abstaining from feeding for several weeks before entering a dormant state. Such measures reduce the risk of ice crystallization in tissues, which is fatal for freezing-intolerant animals. Winter foraging, therefore, may significantly increase the risk of mortality as temperatures drop. Secondly, food availability also limits winter activity. Most fungi likely constituting the snails’ food source (e.g., *Dematiaceae*, *Helminthosporium*, *Macrosporium*, *Alternaria*, and *Ustilago longissima*) are mesophilic, thriving at temperatures between 5 °C and 35 °C [59,60]. However, early spring activity, when temperatures rise but frosts still occur, may allow snails to exploit emerging food resources while maintaining cold resilience.

Cold tolerance is a key factor in determining the geographic range of species [61]. For land snails inhabiting regions with prolonged, harsh winters, surviving low temperatures during overwintering poses a significant challenge. The hypometabolic state serves as a critical strategy to prevent starvation, as both the snails and their food sources remain dormant during this period. However, climate change is expected to result in winters with cold temperatures but lacking insulating snow cover [62]. Experimental studies on survival at low temperatures have demonstrated that snails exhibit limited resilience when snow is absent [29]. This suggests that climate change may significantly impact snail life cycles by reducing their cold tolerance in snowless winters, potentially leading to range contraction in colder regions.

## 5. Conclusions

In summary, our results suggest that temperature is a primary factor limiting the activity and survival of microscopic land snails during winter. Although the ability to remain minimally active at low temperatures may represent an adaptive strategy, it also carries risks, such as interfering with physiological preparations for overwintering. Furthermore, observed shell size variations suggest local adaptations to climatic and environmental conditions. Our study provides new insights into the cold-adaptive strategies of *Vertigo* snails, shedding light on their capacity to cope with winter conditions across diverse climatic zones and habitats.

## Figures and Tables

**Table 1:** Climatic conditions: Comparison of climatic conditions in the two investigated regions, Poznań (Western region) and Kielce (Eastern region), during the decades 1971–1980 and 2011–2020. The parameters include mean air temperature (T mean), standard deviation (SD), minimum air temperature (T min), snow-cover thickness (S), and the number of days with snow cover (Ds). Data sourced from the Institute of Meteorology and Water Management—National Research Institute, [46].

1971–1980														
	Poznań (Western Region)							Kielce (Eastern Region)						
	T Mean	SD	T Min	SD	S [cm]	SD	Ds	T Mean	SD	T Min	SD	S [cm]	SD	Ds
Jan	−1.7	4.8	−4.2	5.6	4.3	6.2	166	−3.5	5.0	−6.6	6.1	7.3	6.7	234
Feb	−0.2	3.6	−2.8	4.1	3.4	7.1	98	−1.4	4.0	−4.4	5.2	5.4	9.0	148
Mar	3.1	4.4	−0.5	4.3	1.1	3.2	52	2.2	4.6	−1.8	4.5	1.9	4.9	71
Apr	6.8	3.2	2.0	3.4	0.1	0.6	6	6.4	3.4	1.6	3.5	0.1	0.8	4
May	12.9	4.2	7.3	4.1	0.0	0.0	0	12.3	4.3	6.6	4.2	0.0	0.0	0
Jun	16.4	3.7	11.1	3.6	0.0	0.0	0	15.4	3.6	9.8	3.5	0.0	0.0	0
Jul	17.7	3.3	12.7	2.9	0.0	0.0	0	16.6	3.0	11.1	3.1	0.0	0.0	0
Aug	17.6	3.2	12.4	3.2	0.0	0.0	0	16.4	3.2	11.0	3.4	0.0	0.0	0
Sep	13.0	3.5	8.6	3.8	0.0	0.0	0	12.0	3.6	7.3	3.6	0.0	0.0	0
Oct	8.0	3.7	4.6	3.9	0.0	0.0	0	6.9	3.9	3.1	4.0	0.0	0.5	2
Nov	3.8	3.7	1.4	3.9	0.2	1.3	18	2.4	4.1	−0.2	4.2	1.0	2.4	70
Dec	0.8	4.4	−1.6	4.9	1.4	2.9	83	−0.7	4.5	−3.4	5.4	2.9	4.6	129
2011–2020														
Jan	0.1	4.6	−2.0	4.8	1.6	3.8	114	−1.9	4.7	−4.6	5.5	4.7	5.8	182
Feb	0.8	5.2	−2.0	5.5	1.0	2.9	50	−0.8	5.3	−4.2	5.9	3.8	5.1	141
Mar	4.4	4.3	0.3	4.7	0.9	3.4	32	3.2	4.3	−1.4	4.5	0.4	1.2	36
Apr	10.0	4.4	4.7	4.0	0.2	1.5	7	9.0	4.3	2.8	4.0	0.2	1.0	12
May	14.5	4.3	8.9	4.2	0.0	0.0	0	13.5	4.0	7.6	3.9	0.0	0.0	0
Jun	18.4	3.6	13.2	3.3	0.0	0.0	0	17.9	3.4	12.0	3.6	0.0	0.0	0
Jul	19.8	3.1	14.6	2.9	0.0	0.0	0	18.9	3.1	13.0	3.4	0.0	0.0	0
Aug	20.0	3.3	14.7	3.3	0.0	0.0	0	19.0	3.4	12.8	3.5	0.0	0.0	0
Sep	15.1	3.3	10.5	3.5	0.0	0.0	0	14.1	3.4	8.9	3.8	0.0	0.0	0
Oct	10.0	3.6	6.5	3.9	0.0	0.0	0	8.6	3.8	4.3	3.9	0.0	0.4	4
Nov	5.3	3.6	2.9	3.8	0.0	0.0	0	4.2	3.9	1.3	4.1	0.1	0.4	12
Dec	2.7	3.6	0.5	3.8	0.4	1.6	27	0.8	3.8	−1.9	4.1	0.7	2.0	65

**Table 2:** The results of the repeated measures Generalized Linear Model showing the effects of the light/dark cycle and temperature on the activity of *Vertigo moulinsiana* individuals collected in winter 2022 (preliminary study).

Variable	F	df1	df2	*p*
Temperature	68.3	2	102	<0.001
Light	0.11	1	102	0.742
Temperature * Light	0.98	2	102	0.377

**Table 3:** The results of repeated measures GLZ models (logit, binomial) showing the effects of temperature, locality, the light/dark cycle, and their interactions (temperature and locality, temperature and light/dark cycle, and locality and light/dark cycle) on the activity of *Vertigo moulinsiana* and *V. antivertigo* individuals.

Variable	*Vertigo moulinsiana*				*Vertigo antivertigo*			
	F	df1	df2	*p*	F	df1	df2	*p*
Temperature	57.3	2	651	<0.001	52.5	2	438	<0.001
Locality	5.9	2	651	0.003	1.0	2	438	0.329
Light	23.9	1	651	<0.001	12.7	1	438	<0.001
Temperature * Locality	2.3	4	651	0.055	2.0	2	438	0.142
Temperature * light/dark cycle	4.4	2	651	0.013	0.3	2	438	0.721
Locality * light/dark cycle	4.5	2	651	0.012	0.08	1	438	0.775

**Table 4:** Basic statistics for SCP values measured in *Vertigo antivertigo* and *V. moulinsiana* collected at each locality during winter (2020 [29] and 2022) and autumn 2022. Localities are abbreviated as follows: S—south, E—east, and W—west.

Time	Locality	*Vertigo antivertigo*					*Vertigo moulinsiana*				
		N	Mean [°C]	Min [°C]	Max [°C]	SD	N	Mean [°C]	Min [°C]	Max [°C]	SD
winter 2020	S	-	-	-	-	-	29	−9.9	−15.0	−6.3	2.2
winter 2022	S	-	-	-	-	-	17	−10.6	−16.7	−5.0	3.7
autumn 2022	S	11	−17.7	−23.5	−15.0	3.0	23	−11.9	−20.7	−7.6	3.6
	E	-	-	-	-	-	34	−11.2	−20.0	−4.2	4.3
	W	8	−11.3	−19.0	−3.7	5.2	24	−10.4	−16.0	−4.6	3.4

**Table 5:** Basic statistics for shell measurements in *Vertigo antivertigo* and *V. moulinsiana* individuals collected at different sites. *V. antivertigo* individuals were not found at the east site.

*Vertigo moulinsiana*												
Variable	Site											
	West N = 9				South N = 7				East N = 12			
	Mean	Min	Max	SD	Mean	Min	Max	SD	Mean	Min	Max	SD
Shell height [mm]	2.41	2.30	2.56	0.08	2.53	2.47	2.69	0.08	2.45	2.47	2.70	0.16
Shell width [mm]	1.51	1.41	1.61	0.07	1.50	1.47	1.54	0.02	1.54	1.44	1.61	0.05
Last whorl width [mm]	1.49	1.42	1.54	0.04	1.55	1.53	1.61	0.03	1.57	1.47	1.61	0.04
Entrance location (R) [mm]	0.74	0.56	0.88	0.10	0.64	0.53	0.72	0.06	0.65	0.60	0.72	0.05
Aperture area [mm^2^]	0.22	0.19	0.26	0.02	0.21	0.18	0.25	0.02	0.24	0.18	0.29	0.04
***Vertigo antivertigo***												
**Variable**	**Site**											
	**West** **N = 14**				**South** **N = 6**				**East** **N = 0**			
	**Mean**	**Min**	**Max**	**SD**	**Mean**	**Min**	**Max**	**SD**	**Mean**	**Min**	**Max**	**SD**
Shell height [mm]	1.91	1.75	2.18	0.12	2.07	1.98	2.16	0.07	-	-	-	-
Shell width [mm]	1.21	1.07	1.30	0.08	1.42	1.21	1.92	0.25	-	-	-	-
Last whorl width [mm]	1.23	1.14	1.33	0.05	1.30	1.24	1.37	0.05	-	-	-	-
Entrance location (R) [mm]	0.56	0.45	0.66	0.07	0.59	0.50	0.68	0.07	-	-	-	-
Aperture area [mm^2^]	0.10	0.08	0.12	0.01	0.12	0.11	0.14	0.01	-	-	-	-

## Data Availability

The original data generated in this study are openly available in FigShare at https://figshare.com/s/e4e3f9e007cad880fb17?file=51421439 (accessed on 30 December 2024).
